# Amyloidogenic Regions and Interaction Surfaces Overlap in Globular Proteins Related to Conformational Diseases

**DOI:** 10.1371/journal.pcbi.1000476

**Published:** 2009-08-21

**Authors:** Virginia Castillo, Salvador Ventura

**Affiliations:** Departament de Bioquímica i Biologia Molecular and Institut de Biotecnologia i de Biomedicina, Universitat Autònoma de Barcelona, Barcelona, Spain; National Cancer Institute, United States of America and Tel Aviv University, Israel

## Abstract

Protein aggregation underlies a wide range of human disorders. The polypeptides involved in these pathologies might be intrinsically unstructured or display a defined 3D-structure. Little is known about how globular proteins aggregate into toxic assemblies under physiological conditions, where they display an initially folded conformation. Protein aggregation is, however, always initiated by the establishment of anomalous protein-protein interactions. Therefore, in the present work, we have explored the extent to which protein interaction surfaces and aggregation-prone regions overlap in globular proteins associated with conformational diseases. Computational analysis of the native complexes formed by these proteins shows that aggregation-prone regions do frequently overlap with protein interfaces. The spatial coincidence of interaction sites and aggregating regions suggests that the formation of functional complexes and the aggregation of their individual subunits might compete in the cell. Accordingly, single mutations affecting complex interface or stability usually result in the formation of toxic aggregates. It is suggested that the stabilization of existing interfaces in multimeric proteins or the formation of new complexes in monomeric polypeptides might become effective strategies to prevent disease-linked aggregation of globular proteins.

## Author Summary

The aggregation of proteins in tissues is associated with the pathogenesis of more than 40 human diseases. The polypeptides underlying disorders such as Alzheimer's and Parkinson's are devoid of any regular structure, whereas the polypeptides causing familial amyotrophic lateral sclerosis or nonneuropathic systemic amyloidosis correspond to globular proteins. Little is known about the mechanism by which globular proteins under physiological conditions aggregate from their initially folded and soluble conformations. Interestingly, several of these pathogenic proteins display quaternary structure or are bound to other proteins in their physiological context. In the present work, we show that protein-protein interaction surfaces and regions with high aggregation propensity significantly overlap in these polypeptides. This suggests that the formation of native complexes and self-aggregation reactions probably compete in the cell, explaining why point mutations affecting the interface or the stability of the protein complex lead in many cases to the formation of toxic aggregates. This study proposes general strategies to fight against diseases associated with the deposition of globular polypeptides.

## Introduction

The formation of insoluble amyloid protein deposits in tissues is related to the development of more than 40 different human diseases, many of which are debilitating and often fatal. The polypeptides responsible for these disorders are not related in terms of sequence or conformation [1]–[6]. Some of these proteins and peptides are mostly unstructured. Examples include amylin, amyloid-β-protein and α-synuclein. In contrast, many other amyloidogenic proteins are globular in their native state, implying that they have a properly packed and cooperatively sustained structure under physiological conditions. This group includes ß-2-microglobulin, transthyretin, lysozyme, superoxide dismutase 1 and immunoglobulins. As a general trend, evolution has endorsed globular proteins with solubility in their biological environments [7]. However, it has been shown that, *in vitro*, under conditions where they become totally or partially unfolded, both these pathogenic proteins [8]–[11] and many globular polypeptides not related to disease [12]–[15] readily convert into aggregates and ultimately into highly structured amyloid fibrils. This self-assembly process is triggered by the destabilization and opening of the native structure, which exposes previously protected aggregation-prone regions that can nucleate the aggregation reaction and participate in forming the β-core of the mature fibril through specific intermolecular interactions [16]–[18]. Such amyloidogenic sequence stretches have been described in most of the polypeptides underlying neurodegenerative and systemic amyloidogenic disorders. The main intrinsic protein properties that promote the assembly of such sequences into fibrils have been recently defined [19], and several algorithms that predict amyloidogenic sequences with good accuracy are already available [3],[20],[21].

Although the study of protein aggregation from non-native states has provided a wealth of data on the physico-chemical determinants of amyloid formation, little is known about how globular proteins aggregate from their initially folded and soluble conformations under physiological conditions, where extensive unfolding is not expected to occur [22]. Deciphering this issue is important because the deposition of globular polypeptides is linked to devastating disorders, and there is an urgent need for therapeutic intervention.

Protein aggregation can be seen as an anomalous type of protein-protein interaction. In functional interactions, binding partners come together in a stable and precise orientation in seconds [23]. This efficiency relies on the structural features of the interacting surfaces. Perhaps the most significant characteristic of a functional protein-protein interface is the presence of small high-affinity regions within the interface, with a reduced number of residues accounting for most of the binding energy [24]–[26]. Several computational approaches have been shown to forecast such regions with high accuracy [27]–[34]. Statistical analysis of the structures of protein-protein interfaces has revealed that tryptophan, phenylalanine, and methionine and to a lesser extent leucine, valine, and tyrosine are preferentially conserved at interaction sites [35]. The same residues have been shown to be conserved in the aggregation-prone sequences of the human proteome [36]. This suggests an intriguing possibility: that amyloidogenic regions and interacting surfaces might overlap in globular proteins. Several of the folded proteins linked to amyloid diseases display quaternary structure or are bound to other proteins in their physiological context. If these interactions specifically cover amyloidogenic regions, they could play a role in protecting native-state proteins from aggregation. Alternatively, incorrect docking of interfaces might facilitate the assembly of overlapping amyloidogenic regions and therefore the formation of toxic protein aggregates of globular proteins. In the present work, we have used available computational approaches to predict aggregation-prone sequences and interacting residues in order to assess the extent to which these regions coincide in pathogenic and non-pathogenic proteins.

## Results/Discussion

### Prediction of Aggregation-Prone Regions and Protein-Protein Interaction Sites

The prediction of regions responsible for aggregation based on the primary sequence of a protein has been tackled by several methods, from simple considerations of the properties of amino acids to complex molecular dynamics calculations [37]–[44]. Overall, most of these methods predict with reasonable precision the regions of proteins in the cross-ß core of amyloid fibrils. This accuracy allows the proposal that the aggregation propensity of a polypeptide chain is ultimately dictated by the sequence [45]. Here we have used four different algorithms in parallel to provide a consensus prediction of the amyloidogenic regions in globular proteins linked to deposition diseases (see Methods). We chose the algorithms implemented by Fernandez-Escamilla *et al.* (TANGO) [38], Conchillo-Sole *et al.* (AGGRESCAN) [40], Galzitskaya *et al.* [41], and Zhang *et al.* [43]. All of them use the primary sequence as input and assume that the detected regions need to be at least partially exposed to solvent in order to nucleate the aggregation reaction.

Identification of binding sites in polypeptides is a direct computational approach to deciphering biological and biochemical function. Although sequence-based approaches to identifying protein interfaces exist, their results are often unsatisfactory. Here, we have used three different structure-based methods whose algorithms are publicly available as web servers to produce a consensus prediction of the interaction interfaces in the globular proteins under consideration (see Methods). These structure-based methods were developed by Fernandez-Recio *et al.* (ODA) [32], Murakami and Jones (SHARP^2^) [31], and Negi *et al.* (InterProSurf) [33]. Although they are based on different principles and implement diverse computational strategies, all of them use the unbound three-dimensional structure of a globular protein as input.

**Figure 1:**
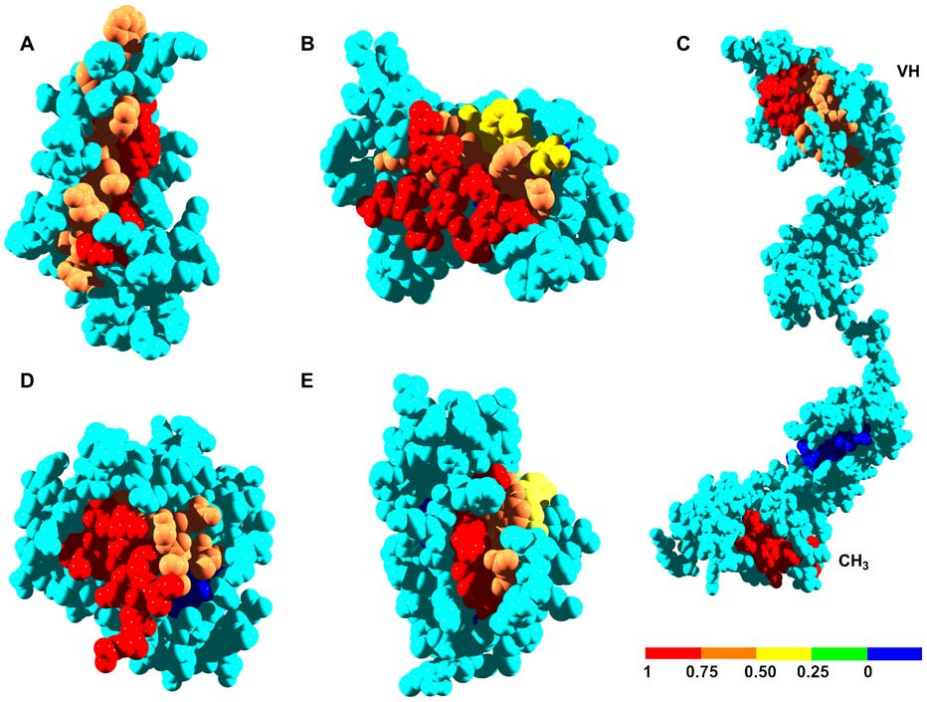
Interface Proximity Index (IPI) of aggregation-prone regions in human globular amyloidogenic proteins. Aggregation-prone regions are coloured according to their IPI values (see the scale). A) β2-microglobulin, B) transthyretin, C) immunoglobulin G heavy chain, D) SOD1 and E) immunoglobulin light chain variable domain.

Two levels of prediction were considered: i) residues predicted or shown to be both in aggregation-prone regions and at interfaces and ii) residues in aggregation-prone sequences that are close in space to the interaction surface (below 3 Å). The interaction predictions were compared with the experimentally determined contacts in the quaternary structure of the proteins or in complexes of the studied proteins with other polypeptides. The regions predicted to have high aggregation propensity were compared with fragments of the analyzed proteins shown experimentally to form amyloid aggregates or to be located in the core of the mature fibrils formed by these polypeptides. We have defined a parameter called Interface Proximity Index (IPI) to evaluate the degree to which an aggregation-prone region is closer to a given interface than to the rest of the protein surface (see Methods and Figure 1).

### Human ß2-Microglobulin

**Figure 2:**
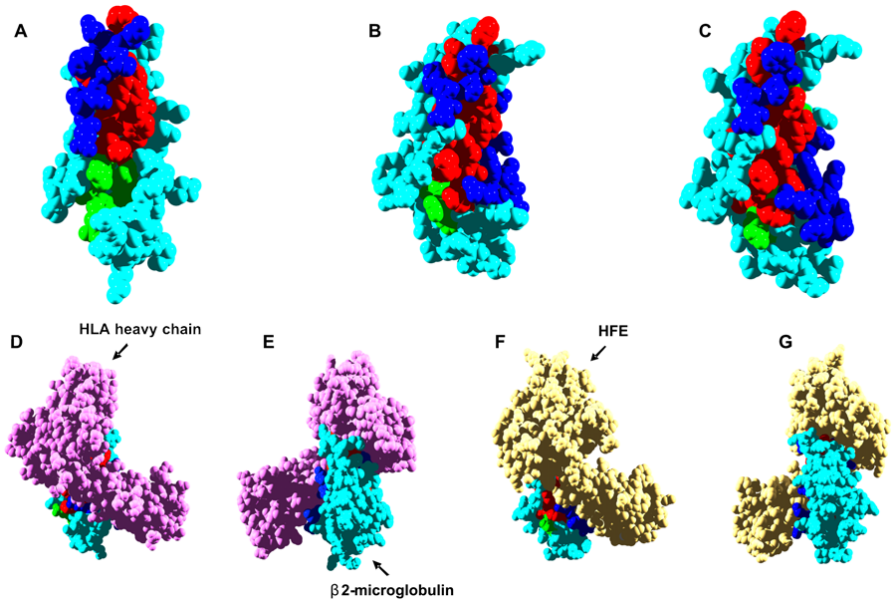
Aggregation and interaction regions in human ß2-microglobulin. In all panels, β2-microglobulin aggregation-prone residues at less and more than 3 Å from interaction sites are shown in red and green, respectively. Interface residues not included in aggregation-prone regions are shown in dark blue. Rest of residues are shown in light blue. A) The predicted interaction surface for monomeric β2-microglobulin is used for calculation. B) The interface between β2-microglobulin and HLA heavy chain is used for calculation (PDB ID:1DUZ). C) The interface between β2-microglobulin and HFE is used for calculation (PDB ID:1A6Z). D and E) Front (same orientation that in B) and back view of the β2-microglobulin/HLA heavy chain complex. F and G) Front (same orientation that in C) and back view of the β2-microglobulin/HFE complex.

Amyloidosis related to β2-Microglobulin (β2-m) is a common and serious complication in patients on long-term hemodialysis [46]. Two aggregation-prone regions encompassing residues 22–31 and 60–70 were predicted for human β2-m (Figure 2). These regions neatly coincide with two secondary structure elements in β2-m: β-strand 2, formed by residues 21–31, and β-strand 6, formed by residues 61–71. Interestingly, most of the residues in these two regions appear to be solvent accessible (Table 1). In agreement with the prediction, the fragments 21–31 and 21–41 of β2-m self-assemble into fibrillar structures [47]. Also, a peptide corresponding to residues 59–79 and its shorter version 59–71 both form amyloid fibrils [48].

**Table 1:** Comparison of aggregation predictions and experimental available data for human globular proteins and proximity of aggregation-prone regions to predicted and real interfaces.

Predicted Aggregation segments	Fibril formers	% residues close to predicted Interface^1^	% residues close to real interface^2^	IPI	% solvent accessible residues
**ß2-Microglobulin (HLA)**					
22–31	21–31	70 (20)	100 (18)	0.88	65
	21–41				
60–70	59–79	54 (24)	73 (30)	0.58	100
	59–71				
**ß2-Microglobulin (HFE)**					
22–31	21–31	70 (20)	70 (12)	0.83	65
	21–41				
60–70	59–79	54 (24)	81 (31)	0.62	100
	59–71				
**Transthyretin**					
11–19	10–20	44 (22)	77 (36)	0.46	66
26–34	-	0 (53)	0 (68)	<0	66
92–96	-	40 (42)	100 (0)	1	100
105–112	105–115	62 (30)	100 (40)	0.6	100
115–121	-	62 (32)	100 (0)	1	100
**SOD1**					
4–8	-	0 (13)	100 (3)	0.97	66
100–106	-	43 (12)	0 (19)	<0	66
111–120	-	70 (18)	50 (14)	0.72	100
146–153	-	87 (21)	87 (2)	0.97	100
**Lysozyme**					
25–33	-	33	0 (25)	<0	44
57–66	-	90	40 (32)	0.20	60
76–84	26–123	56	56 (25)	0.55	88
108–114	-	86	0 (46)	<0	100
**Immunoglobulin (LC)**					
19–23	-	-	0 (38)	<0	71
31–38	-	-	89 (24)	0.71	75
46–51	-	-	50 (30)	0.4	71
71–78	-	-	0 (43)	<0	62
84–89	-	-	83 (18)	0.78	66
**Immunoglobulin (HC)**					
29–38	-	-	50 (20)	0.60	80
45–52	-	-	75 (25)	0.67	82
87–93	-	-	57 (24)	0.58	57
100–106	-	-	100 (16)	0.84	100
275–281	-	-	0 (31)	<0	71
289–299	-	-	0 (52)	<0	100
322–331	-	-	0 (37)	<0	80
390–396	-	-	63 (13)	0.79	57
435–442	-	-	100 (16)	0.84	75

1 Percentage of residues in the aggregation-prone region at less than 3 Å from a protein predicted interaction residue.

2 Percentage of residues in the aggregation-prone region at less than 3 Å from a residue located at the interface of the following complexes: β2-microglubulin in complex with HLA heavy chain [1DUZ] and with HFE [1A6Z]. Native tetrameric structure of transthyretin (PDB code 1TTA). Dimeric structure of SOD1 (PDB code 2C9V). Lysozyme in complex with a camelid antibody (PDB code 1OP9). Dimeric structure of Immunoglobulin LC variable domain (PDB code 2Q20). HCs and LCs of a IgG1 human immunoglobulin (PDB code 1HZH).

1,2 In brackets the percentage of residues in the aggregation-prone region close to a random surface of the same size than the considered interface.

A main interaction cluster is predicted for human β2-m (Figure 2A). It involves Y26 and G29 in β-strand 2, residues H31-S33 in the loop connecting β-strands 2 and 3, residues D53-W60 in β-strand 5 and the adjacent loop, and finally, residues F62 and L63 in β-strand 6. Overall, 62% of the residues in regions with high aggregation propensity are less than 3 Å from predicted “hot spots” of interaction (Table 1), and 25% overlap with them. Specifically, residues at positions 26, 29, 31, 60, 62, and 63 are predicted to be important both for binding and for aggregation.

Class I major-histocompatibility-complex (MHC) molecules (HLA molecules in humans) are ternary complexes of β2-m, an MHC heavy chain, and a bound peptide [49]. The crystal structures of several of these complexes have been solved, providing a benchmark to evaluate the accuracy of the predicted interface. In HLA-A-class molecules, the interface of β2-m and the HLA heavy chain is well conserved [50] and typically comprises 16 β2-m residues: K6, Q8, 10Y, 11S, 12R, N24, Y26, H31, D53, S55, F56, W60, F62, Y63, D98 and M99. This includes 8 of the 15 interacting residues predicted for β2-m. Residues 24, 26, and 31 map to the first aggregation-prone region of β2-m, and residues 60, 62, and 63 map to the second one. Taking as an example the structure of one such HLA-A complex (PDB ID: 1DUZ) [51], 85% of the residues in β2-m aggregating regions are less than 3 Å from the interface in the complex (Table 1 and Figures 2B, 2D and 2E). The IPI values confirm that these regions are preferentially located close to the interface of the complex (Table 1 and Figure 1A).

Inside the cell, β2-m associates with the non-classical HLA class I molecule human hemochromatosis protein (HFE) [52]. Hereditary hemochromatosis is a genetic disorder characterized by defects in iron metabolism and associated with mutations in the HFE gene [53]. Some of these mutations prevent the binding of HFE to β2-m. There are 18 β2-m residues at the HFE/β2-m complex interface, according to its crystal structure (PDB ID: 1A6Z) [54] : I1, Q8, 10Y, 11S, 12R, N24, Y26, H31, D53, L54, S55, F56, W60, Y63, F62, L65, D98, and M99, including 9 of the 15 predicted interaction sites. Residues 24, 26, and 31 correspond to the first aggregation-prone region of β2-m and residues 60, 63, 62, and 65 to the second. Another significant feature of this complex is that 76% of the residues in regions with high aggregation potential are close to the interface with β2-m (Table 1 and Figure 2C). Therefore, the docking of the HLA heavy chain and HFE molecules on top of β2-m covers most of the residues in aggregation-prone regions because they are close to the interaction sites, as illustrated by their high IPIs (Table 1, Figure 1A and Figures 2F, 2G).

Aggregation of β2-m under physiological conditions is thought to be initiated by a cis-trans prolyl isomerization of the H31-P32 peptide bond [22]. The transition promotes repositioning of the hydrophobic side chains of F30, L54, F56, W60, F62, and Y63 as shown in the structures of the P32A and P32G mutants [55],[56]. Interestingly enough, all of these residues map in an aggregation-prone segment and/or at the interface. Although speculative, it is tempting to propose that conditions that promote the dissociation of β2-m complexes with the above proteins or related ones may uncover this region and facilitate its fluctuation towards amyloidogenic conformations. In fact, *in vivo*, β2-m is continuously shed from the HLA molecules in the cell surface into the serum and transported to the kidneys where it is eliminated. Renal failure increases the levels of circulating β2-m more than 50-fold and promotes its self-assembly and conversion into amyloid fibrils [57]. Consequently, dissociation of β2-m from the class I HLA complex effectively constitutes a critical initial step in its aggregation into amyloid fibrils.

Because the β2-m regions likely to be involved in aggregation are already located in preformed β-strands, local fluctuations may allow anomalous intermolecular interactions between these preformed elements, leading to the formation of an aggregated β-sheet structure without extensive unfolding. In this context, the formation of β2-m complexes both inside the cell and on the cell surface might play a protective role against β2-m aggregation, either by reducing conformational fluctuations or by preventing the exposure of dangerous amyloidogenic regions, or both.

### Human Transthyretin

Transthyretin (TTR) constitutes the fibrillar protein found in familial amyloidotic polyneuropathy (FAP), familial amyloidotic cardiomyopathy, and central nervous system amyloidosis. Around 100 different TTR mutations have been reported, many of which are amyloidogenic [58]. Native TTR is a homotetramer. Five aggregation-prone regions are predicted for the TTR monomer. They encompass residues 11–19, 26–34, 92–96, 105–112, and 115–121. In this case, the aggregation-prone sequences appear to coincide precisely with preformed β-sheet structures: A β-strand (11–19), B β-strand (26–36), F β-strand (91–97), G β-strand (105–112), and H β-strand (115–121). In concordance with the prediction, peptides 10–20 and 105–115, which map in the first and fourth aggregation-prone regions, have been shown to assemble into amyloid fibrils [59],[60].

**Figure 3:**
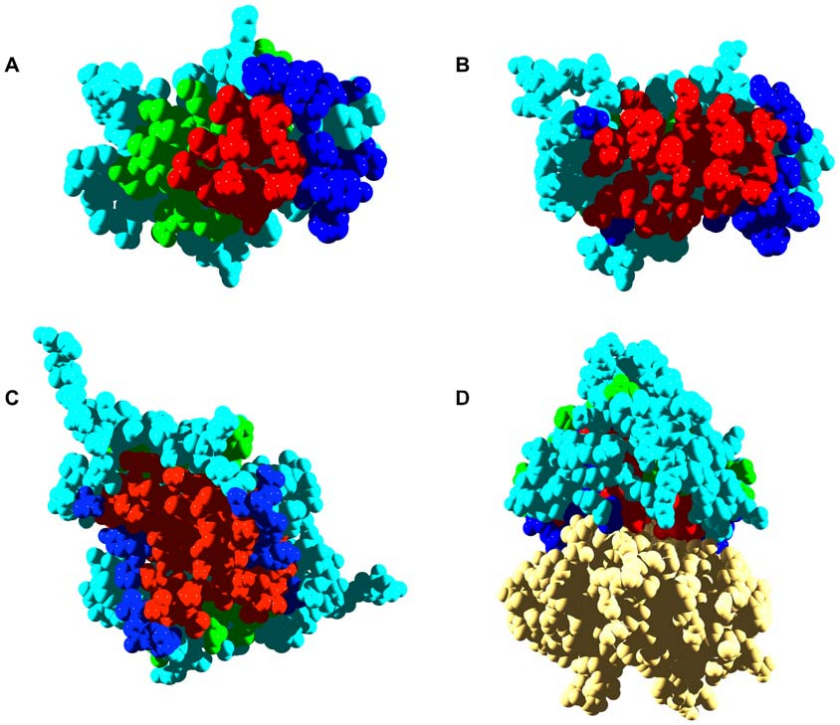
Aggregation and interaction regions in human transthyretin. In all panels, transthyretin (TTR) aggregation-prone residues at less and more than 3 Å from interaction sites are shown in red and green, respectively. Interface residues not included in aggregation-prone regions are shown in dark blue. Rest of residues are shown in light blue. A) The predicted interaction surface of a TTR monomer is used for calculation. B) The interface in the native tetrameric structure of TTR is used for calculation (PDB ID:1TTA). C) Dimer of TTR. D) TTR native tetrameric structure. The first dimer is twisted 90° relative to C, the second one is shown in yellow.

A single interaction patch is predicted for the TTR monomer (Figure 3A). It involves 19 residues located in the A β-strand (L17, A19), in the loop between the A and B β-strands (V20–S23), in the α-helix (L82), in the loop between the helix and the F β-strand (S85-F87), in the F β-strand (E92), in the G and H β-strands, and in the loop between the G and H β-strands (L110, S112-T118). TTR is a dimer of dimers. In the dimers formed by the A and B or the C and D chains, the predicted clusters are contiguous, forming a large and continuous interaction patch. Of the residues in aggregation-prone regions in TTR, 41% are within 3 Å of predicted interaction sites (Table 1). With the exception of the I26-R34 fragment, all the regions with high aggregation propensity are located close to the predicted interface, and 30% of the residues in these segments overlap with predicted interaction sites. Residues 17, 19, 92, 110, 112, and the stretch 115–118 are predicted to be important both for aggregation and interaction events.

The crystal structure of the TTR tetramer (PDB ID: 1TTA) [61] reveals that the real interfaces between the four individual TTR chains involve residues L17, A19-S23, F87-E89, E92, V94-T96, Y105, L110, and S112-V122. In good agreement with the prediction, the interfaces include 16 of the 19 predicted interacting residues. Residues 17 and 19 map to the first aggregation-prone region, residues 92 and 94–96 to the third one, and residues 110 and 112–122 to the fourth and fifth stretches. Significantly, if we exclude the I26-R34 region (IPI<0), 90% of the residues in aggregating regions are close to the two interfaces of the TTR tetramer as confirmed by their overall high IPIs (Table 1, Figure 1B and Figures 3B, 3C). Accordingly, although these regions are mostly accessible to solvent in the monomer, they become protected in the native quaternary structure of TTR by the interaction of the TTR subunits (Figure 3D).

Dissociation of the TTR tetramer has been reported as a prerequisite for amyloidosis. The tetrameric structure dissociates into AB and CD dimers, but they are unstable in the absence of additional quaternary interactions, explaining why TTR exists in a primarily tetramer-monomer equilibrium [62]. The crystal structures of more than 10 FAP-related variants have been solved, showing that the mutants are essentially identical in tertiary and quaternary structure to the wild-type protein, precluding the presence of preformed conformational defects in the amyloidogenic mutants [63]. However, FAP-associated mutants are destabilized even when tetrameric. This destabilization favors tetramer dissociation to the amyloidogenic monomeric intermediate, exposing previously hidden, preformed, aggregation-prone β-strands. In this context, the overlap of interaction and aggregation surfaces in the AB and CD dimers appears to be an effective way to prevent TTR amyloidogenesis in physiological conditions. The success of this strategy is best exemplified by the behavior of the T119M TTR mutant. The presence of the T119M allele alleviates the effect of the aggressive V30M amyloidogenic mutation in patients carrying these two variants. It has been shown that heterotetramers that incorporate T119M subunits are more stable, dissociate at lower rates, and accordingly are less amyloidogenic [64].

### Human Copper-Zinc Superoxide Dismutase

Familial amyotrophic lateral sclerosis (fALS) is characterized by the presence of Copper-Zinc Superoxide Dismutase (SOD1) inclusions in spinal cords [65]. Native SOD1 is a homodimer. The SOD1 monomer displays four regions with high aggregation potential. They encompass residues 4–8 in ß-strand 1, 100–106 and 111–120 in ß-strands 6 and 7 and the loop connecting them, and residues 146–153 in ß-strand 8.

**Figure 4:**
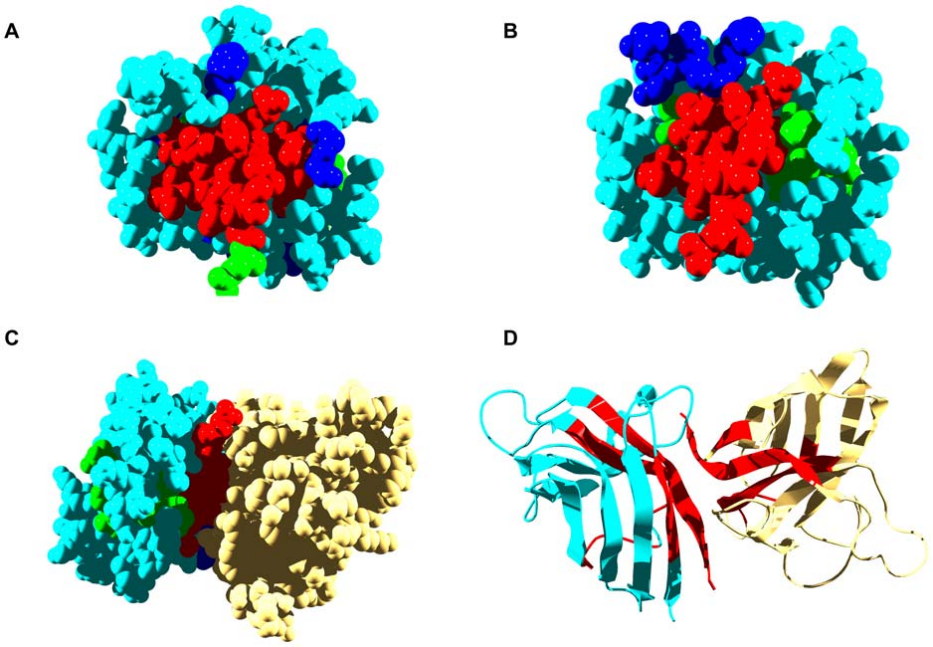
Aggregation and interaction regions in human SOD1. In panels A, B and C SOD1 aggregation-prone residues at less and more than 3 Å from interaction sites are shown in red and green, respectively. Interface residues not included in aggregation-prone regions are shown in dark blue. Rest of residues are shown in light blue. A) The predicted interaction surface of a SOD1 monomer is used for calculation. B) The interface in the native dimeric structure of SOD1 is used for calculation (PDB ID:2C9V). C) Native dimer of SOD1, the second monomer is shown in yellow. D) Ribbon representation of the SOD1 dimer, predicted aggregation-prone regions are shown in red.

A total of 14 residues are predicted to be at the interface of the SOD1 monomer (Figure 4A). They correspond to E21, W32, G33, S105, S107, G108, H110, C111, I113-R115, G147, V148, and I151. Of the residues in aggregation-prone regions in SOD1, 61% are less than 3 Å from predicted interaction sites (Table 1), and 25% of them overlap the predicted interaction sites. In particular, residues 105, 111, 113, 114, 115, 147, 148, and 151 are predicted to be involved in both binding and aggregation.

According to the crystal structure of the SOD1 dimer (PDB ID: 2C9V) [66], the real interface between the two SOD1 subunits involves residues V5, V7, F50-T54, I113-R115, V148, and G150-Q153 (Figure 4B). Therefore, the interaction prediction is poor for the N-terminal part of SOD-1 but accurate for residues in the C-terminal region. Residues V5 and V7 are part of the first aggregation-prone region, S105 part of the third one, I113-R115 part of the fourth stretch, and V148 and G150-Q153 part of the last one. All the residues in the first and last aggregation-prone segments as well as residues C111-T116 are close to the dimer interface (Table 1). Accordingly, except for the 100–106 stretch (IPI<0), all the regions with high aggregation propensity in SOD display high IPIs (Table 1 and Figure 1D). Three out of the four cysteine residues in each SOD1 monomer (6, 111, and 146) are in those sequence stretches. C6 and C111 are present in the form of free cysteines whereas C146 forms a disulfide bond with C57. All of these regions are accessible to solvent in the monomeric form but become partially or totally protected upon dimer association (Figure 4C and 4D).

FALS has been shown to be associated with more than 100 different SOD1 mutations, which are scattered throughout the three-dimensional structure [67]. Among them, the A4V mutation has received special attention because it results in a rapidly progressing form of fALS [68]. Animal models suggest that the pathogenicity of the A4V SOD1 arises from an increased propensity to aggregate, forming amyloid fibrils or pores [69]. A4 is near the dimer interface and maps in the first aggregation-prone region. Hasnain and co-workers solved the crystal structures of dimeric forms of A4V and another FALS mutant, I113T [70]. I113 is also at the interface, in the third aggregation-prone region. Both variants display the same monomer fold and active-site geometry as WT, but their interfaces are destabilized. Ray and Lansbury have shown that a covalent link between the two A4V SOD1 subunits abolishes aggregation, suggesting that the monomer is an obligate intermediate along the aggregation pathway [71]. Other studies also support the idea that monomerization leads directly to aggregation and fibrilization [72]. However, other lines of evidence suggest that the cytotoxic properties of SOD1 are triggered by an incorrect connection of its cysteine residues. In support of this view, the toxicity of recombinant SOD1 in cultured cells is lost upon mutational removal of C6 and C111 [11], and nucleation of the aggregation reaction requires the presence of cysteine thiolates at both positions 57 and 146 [72]. In any case, it appears that the interface plays a protective role against aggregation in SOD1, by preventing the direct assembly of pre-formed and exposed aggregation-prone regions in the monomer, by stabilizing the monomer against conformational fluctuations that might expose amyloidogenic sequences, or by preventing the exposure and reshuffling of cysteine residues. Based on these observations, it has been proposed that the stabilization of the SOD1 dimer interface could become an effective approach to fight against fALS [71].

### Human Immunoglobulins

**Figure 5:**
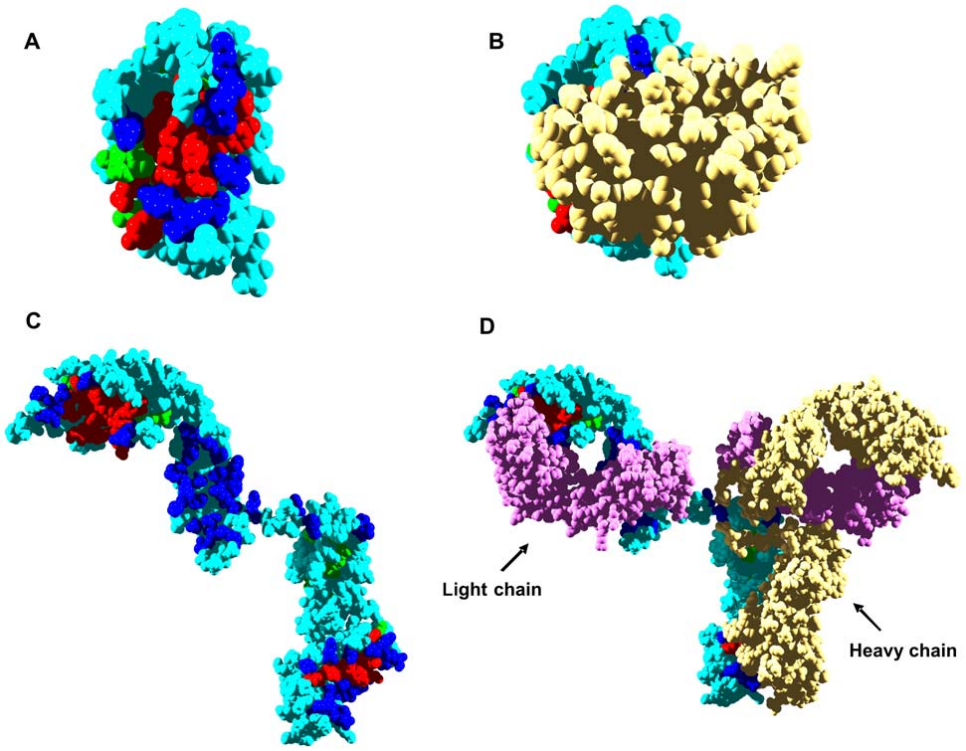
Aggregation and interaction regions in human immunoglobulins. In all panels, immunoglobulin (Ig) aggregation-prone residues at less and more than 3 Å from interaction sites are shown in red and green, respectively. Interface residues not included in aggregation-prone regions are shown in dark blue. Rest of residues are shown in light blue. A) The interface in the native structure of Ig light chain variable domain (LC) is used for calculation (PDB ID: 2Q20). B) Native homodimer of Ig LC, the second monomer is shown in yellow. C) The interface in the native structure of IgG heterotetramer is used for calculation and the Ig heavy chain (HC) represented (PDB ID: 1HZH). D) Native IgG heterotetramer. Ig LCs and the second Ig HC are indicated.

The light chains (LCs) of immunoglobulins have been implicated in the pathogenesis of amyloidosis in patients with monoclonal B-cell proliferative disorders (AL amyloidosis) [73]. When immunoglobulin molecules are secreted, two heavy chains (HCs) usually pair with two LCs to create a heterotetramer. Occasionally, free LCs are secreted, and these LCs can form homodimers. LC dimers can be innocuous, but they can also aggregate into pathogenic species. We have analyzed the aggregation propensity and interfaces of a non-pathogenic LC dimer (PDB ID: 2Q20) [74]. Five aggregation-prone regions are detected, encompassing residues 19–23, 31–38, 46–51, 71–78, and 84–89 located in the ß3, ß4 ß5, ß9, and ß10 strands, respectively (Table 1). The interface of the dimer involves 13 residues: D34, Y36, Q38, K42-P44, L46, E55, Y87, Q89, Y91, Y96, and F98. According to their IPIs, the second and fifth stretch are located preferentially at the interface of the complex, with 89% and 83% of their residues less than 3 Å from the interface, respectively (Table 1, Figure 1E and Figures 5A, 5B). It is important to note that both stretches map in preformed ß strands.

AL is distinct from other types of amyloidosis in that hypervariability yields a different set of mutations in each patient. Ramirez-Alvarado and co-workers have characterized an LC dimer isolated from an AL patient [74]. The pathogenic protein differs from its germline in seven residues. Only three changes are non-conservative, and all of them are located at the dimer interface: N34I, K42Q, and Y87H. The N34I and Y87H mutations occur precisely in the second and fifth aggregation prone regions in the protein. Ramirez-Alvarado and co-workers found that the mutant dimer has an interface that is rotated 90° from the canonical LC interface. The altered interface was accompanied by decreased thermodynamic stability of the dimer and accelerated fibril formation. This might result from the exposure and self-assembly of the above preformed aggregation-prone ß segments upon dimer destabilization or dissociation. Interestingly, the restorative mutation H87Y suffices to regain thermodynamic stability, delay amyloid formation, and restore the canonical dimer interface, illustrating a delicate balance between native and aberrant protein self-assembly.

Although AL is more frequent, in some systemic amyloidosis the amyloid deposits consist of an unusual form of IgG1 heavy chain (HC) [75]. The amyloid protein contains the complete heavy-chain variable (VH) domain contiguous to the third constant region (CH_3_) due to the total absence of the first (CH_1_) hinge and second (CH_2_) heavy-chain constant regions [75].

Using the structure of a complete human IgG1 antibody [76] as a model (PDB ID: 1HZH), we detected nine aggregation-prone regions in the heavy chain (Table 1). Four of the aggregation-prone regions are in the VH domain (29–38, 45–52, 87–93, and 100–106), three in the CH_2_ domain (275–281, 289–299, and 322–331), and two in the CH_3_ domain (390–397 and 435–442). Analysis of the structure of the oligomeric form of the antibody reveals that only the regions in the VH and CH_3_ domains of the heavy chain display high IPI values and therefore are adjacent to the interface in the native heterotetramer (Table 1, Figure 1C and Figures 5C, 5D). The truncated, pathogenic form of the IgG is found in monomeric form in urine, indicating that either it cannot associate or it dissociates from the light and heavy chains that block the exposure of the detected aggregating regions in a normal heterotetrameric IgG molecule. These sequence stretches are located in preformed ß strands and are ready for self-assembly reactions that might result in the observed amyloid deposits.

### Protein Binding Prevents Aggregation: Human Lysozyme and Aß42

Human lysozyme forms amyloid fibrils in individuals suffering from nonneuropathic systemic amyloidosis. The disease is always associated with non-conservative point mutations in the lysozyme gene [77]. Four aggregation-prone regions were detected in human lysozyme, corresponding to residues 25–33, 57–66, 76–84, and 108–114. The first region maps in helix B, the second and third in the loop of the β-domain, and the last one around the short helix D (Table 1). In good agreement with the predictions, recent experimental data shows that the region comprising residues 26–123 is preferentially protected from proteolysis once it is incorporated into lysozyme amyloid fibrils [78].

**Figure 6:**
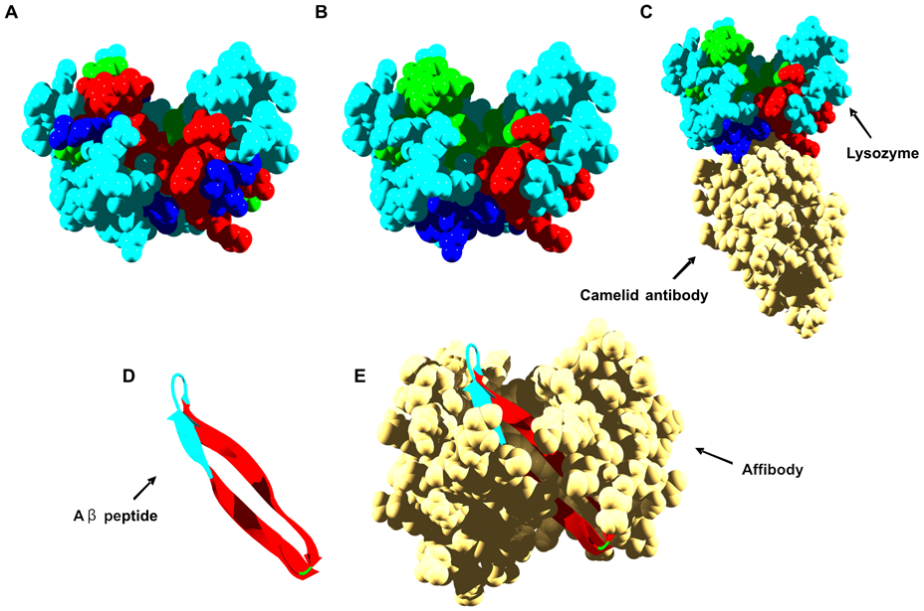
New interfaces at human lysozyme and Aß peptide aggregation-prone regions. In all panels, aggregation-prone residues at less and more than 3 Å from interaction sites are shown in red and green, respectively. Interface residues not included in aggregation-prone regions are shown in dark blue. Rest of residues are shown in light blue. A) The predicted interaction surface of lysozyme is used for calculation. B) The interface between lysozyme and a camelid antibody is used for calculation (PDB ID: 1OP9). C) Lysozyme complex with a camelid antibody. D) Ribbon representation of Aß peptide. The interface between the peptide and a designed affibody is used for calculation (PDB ID: 2OTK). E) Aß peptide bound to a designed affibody.

Two different interaction clusters are predicted for human lysozyme (Figure 6A), one in the α-domain and the other in the ß-domain. The first involves residues in the loop of the β-domain: N60, R62-W64, N66, A73-N75, A76, and H78. The second cluster is located in helix C and around helix D and corresponds to residues A94, K97, R98, R107-W109, and W112. Residues K33 and W34 in helix B are also predicted to be involved in protein-protein interactions. Overall, 66% of the residues in regions with high aggregation propensity are less than 3 Å from predicted interaction sites, and 31% overlap with them. Residues 33, 60, 62–64, 66, 76, 78, 108, 109, and 112 might be implicated in both binding and aggregation reactions. Interestingly, residues I56, F57, W64, and D67, which are mutated in the four known single-residue familial variants associated with lysozyme amyloidosis, are comprised of or very close to protein segments with high aggregation propensity and/or interaction sites.

The mechanism of lysozyme aggregation under physiological conditions probably involves thermal fluctuations that transiently expose amyloidogenic regions [22]. These transitions are rare in the wild type protein, but they are more frequent in mutated forms related to amyloidosis. It has been suggested that residues 36–102 in the β-domain and helix C can unfold while the rest of the α-domain maintains a native-like conformation [9]. In particular, residues 78–80 have been proposed to have a high aggregation propensity and the lowest structural protection, and therefore the highest propensity to initiate aggregation [79]. This sequence includes predicted interacting residues in the loop of the ß-domain and also overlaps with the predicted 76–84 amyloidogenic region.

A single-domain fragment of a camelid antibody has been shown to inhibit the *in vitro* aggregation of the D67H amyloidogenic lysozyme variant [80]. The antibody epitope includes neither the site of mutation nor most of the protein region destabilized by the mutation; therefore it was suggested that the binding of the antibody prevents aggregation by restoring the structural cooperativity of the mutant protein through the transmission of long-range conformational effects [80]. The structure of the antibody-lysozyme complex (PDB ID: 1OP9) reveals that the epitope consists of 14 residues of the lysozyme molecule and encompasses residues located in the loop between the A and B helices in the α-domain (L15, G16, Y20), in the long loop within the ß-domain (A76, C77, H78, L79), and in the C-helix (A90, D91, A94, C95, K97, R98, R101) (Figure 6B). The epitope includes interaction residues in the first and second predicted clusters. Also, the residues in the loop of the ß-domain coincide with the 76–84 aggregation-prone region. Therefore, an alternative explanation for the protective action of the antibody could be that by docking on top of interaction clusters, it impedes the conformational fluctuation and exposure of the amyloidogenic region around residues 70–80 (Figure 6C).

A nice example illustrating how new binding interfaces can effectively inhibit amyloid formation has been recently reported for the Alzheimer's Aβ peptide. Two aggregation-prone regions comprising residues 16–21 and 29–40 are consistently predicted for Aβ (Figure 6D). The prediction is in excellent agreement with the experimental data in the literature indicating that these regions constitute the core of the Aβ fibrils [81]. Härd and co-workers have used the Z domain derived from staphylococcal protein A to evolve variants of this domain able to bind to Aβ with nanomolar affinity and abolish its aggregation (affibodies) [82]. The solution structure of one of these complexes illustrates how the affibody's protective effect is exerted by creating a new, continuous interface with Aβ that buries its two aggregation-prone regions within a large hydrophobic tunnel-like cavity (Figure 6E).

### Non-Amyloidogenic Monomeric Proteins

An important question to address is whether predicted interaction interfaces and aggregation-prone regions also coincide in monomeric and soluble proteins. Therefore, we have analyzed the predicted properties of four well-characterized soluble proteins: myoglobin, maltose binding protein, thioredoxin, and ubiquitin.

**Figure 7:**
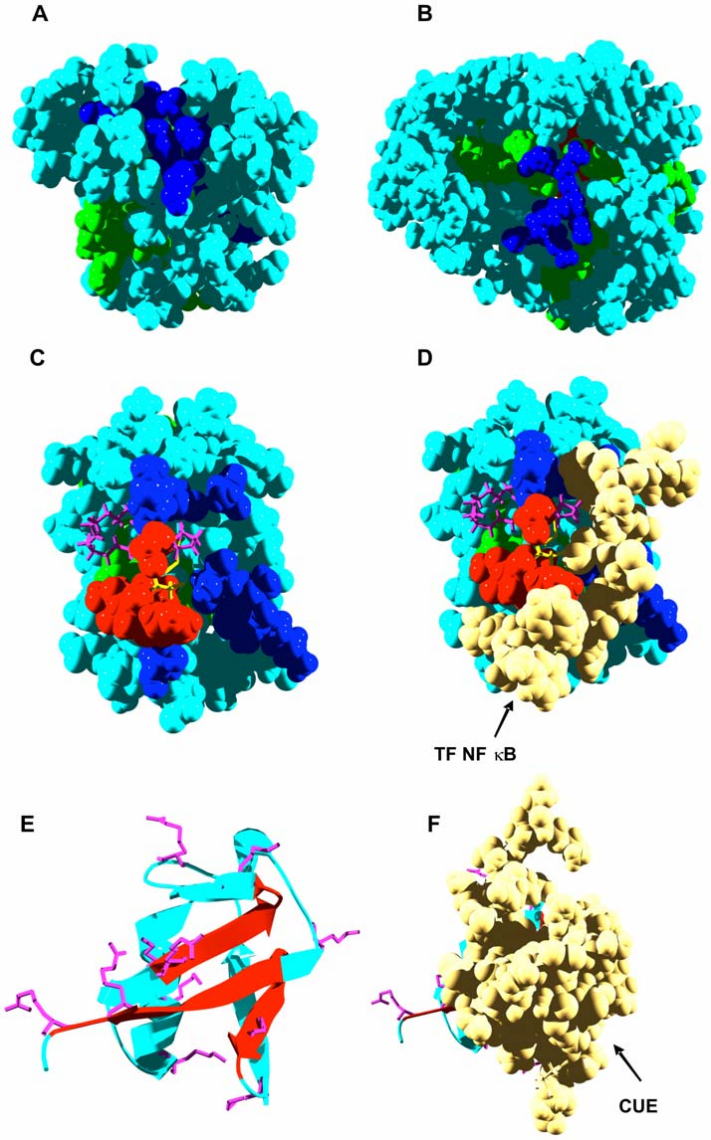
Aggregation and interaction regions in monomeric soluble proteins. In panels A–D, aggregation-prone residues at less and more than 3 Å from interaction sites are shown in red and green, respectively. Interface residues not included in aggregation-prone regions are shown in dark blue. Rest of residues are shown in light blue. In all panels the predicted interaction surface is used for calculation. A) Human myoglobin (PDB ID: 4MBN). B) Maltose Binding Protein (MBP) (PDB ID: 4MBP). C) Human thioredoxin (TRX) (PDB ID: 3TRX). Gatekeeper residues are shown in purple and active cysteines in yellow. D) Same orientation that in C, Human TRX in a mixed disulfide intermediate complex with a peptide from the transcription factor NF kappa B (PDB ID: 1MDI). E) Ribbon representation of human ubiquitin (PDB ID: 1UBQ). Aggregation-prone secondary structures near the interface are shown in red. Basic residues in the vicinity of aggregation-prone regions are shown in purple. F) Same orientation than E). Complex of human ubiquitin with a CUE ubiquitin binding domain (PDB ID: 1OTR).

Human myoglobin is a compact protein not related to disease. Although after long exposure to high temperatures *in vitro* it unfolds and assembles into amyloid fibrils [15], it is a highly soluble protein in its native α-helical conformation. It displays four regions with high aggregation potential encompassing residues 8–15, 28–33, 67–76, and 110–117. This last segment partially overlaps with the peptide fragment 100–114 found to form amyloid structures *in vitro* [83]. A 12-residue interface is consistently predicted for myoglobin. It consists of residues L40, K42, F43, L89, S92, I99, P100, K102, Y103, I107, L137, and F138. Interestingly enough, only one residue (I111) in the predicted aggregating regions is close to the interface. In addition, its side chain is buried, resulting in a surface where predicted interaction and aggregation regions do not overlap (Figure 7A), a feature that might have evolved to resist aggregation.

Maltose binding protein (MBP) endows fused proteins with increased solubility indicating that it is by itself highly soluble [84]. However, because it is a relatively large protein (370 residues), 10 different aggregation prone regions are predicted, comprising a total of 82 residues. Similarly to the case of myoglobin, although 8 of these residues are close to the predicted interface, comprising residues F92, E153, F156, M321, E322, A324-I329 and W340, their side chains are not significantly exposed to solvent (Figure 7B).

Thioredoxin A (TRX) is another tag used to increase the solubility of recombinant proteins [85]. Three aggregation-prone regions comprising residues 22–27, 29–33, and 49–57 are detected in human TRX. The predicted interaction surface comprises residues T30-I38, D60, V71-T74, and A92. While the first and third aggregation stretches are at more than 3 Å of the predicted interface, the second one overlaps with it. Surprisingly, in contrast to myoglobin and MPB, this region is exposed to solvent (Figure 7C). This suggests that, as discussed in the previous section, it could be involved in protein assembly reactions. In fact, residues C32–C35 in this stretch constitute the consensus CXXC motif in the TRX active site. In agreement with this hypothesis, we found that in the solution structure of human TRX in a mixed disulfide intermediate complex with its target peptide from the transcription factor NF k-B, the second aggregation-prone region in TRX is part of the complex interface [86] (Figure 7D).

The question arises of why TRX does not self-assemble when it is free. It appears that evolution uses negative design to fight against protein deposition by placing amino acids that counteract aggregation at the flanks of protein sequences with high aggregation propensity [45]. These residues are called aggregation gatekeepers [87], and they reduce self-assembly using the repulsive effect of charge (Arg, Lys, Asp and Glu), the entropic penalty on aggregate formation (Arg and Lys), or incompatibility with ß-structure backbone conformation (Pro) [88]. Interestingly, P34 is adjacent in sequence to the TRX 29–33 aggregation prone region. P34 and the two basic, protruding K37 and K39 residues flank this region in the 3D-structure (Figure 7C), which overall would make self-assembly reactions far more difficult.

Ubiquitin is a small, soluble and highly conserved regulatory protein that is ubiquitously expressed in eukaryotes [89]. Three aggregation-prone regions are detected in ubiquitin, including residues 1–8, 42–47, and 67–74 in the ß1, ß3, and ß5 strands, respectively. In this case, the regions of the protein with the highest aggregation propensity overlap significantly with the predicted interaction interface (Figure 7E). This suggests that in principle, this surface is competent for protein assembly reactions. Importantly, it has been shown that ubiquitin binding motifs, such as CUE domains, bind precisely to a surface defined by the ß1, ß3, ß4, and ß5 strands of ubiquitin (Figure 7F) [90], illustrating again how aggregation-prone regions and interaction interfaces tend to overlap. In fact, biochemical and genetic analyses have defined the hydrophobic patch formed by the side chains of L8, I44, and V70 on the surface of ubiquitin as a key determinant for endocytosis and proteosomal degradation [91]. These three residues are located in each of the three aggregation-prone regions predicted for ubiquitin. Why, then, does ubiquitin not self-assemble when it is unbound in solution? An examination of the surface defined by the above ß-strands shows that ubiquitin uses negative design principles to avoid aggregation, placing a large number of positively charged residues on the edge of these strands and adjacent to them (Figure 7E). Upon binding to ubiquitin-binding domains, these basic residues are hidden at the complex interface.

### Non-Amyloidogenic Dimeric Proteins

**Figure 8:**
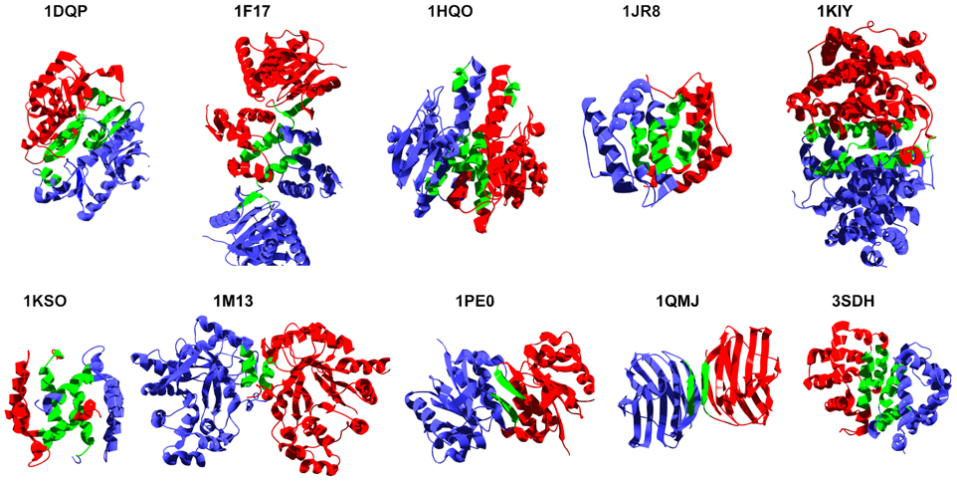
Aggregation-prone regions at the interface of selected homodimeric eukaryotic proteins. Aggregation-prone regions in which more than 85% of the residues are at less than 3 Å from the interface are highlighted in green. The PDB ID is indicated for each dimer (see also Table 2).

It seems that the spatial coincidence of interfaces and sequences promoting self-assembly is not restricted to amyloidogenic proteins. To further confirm this extent, we analyzed the structure of 25 different eukaryotic proteins shown to form homodimers (Table 2 and Figure 8). As expected, the number of predicted aggregation-prone regions in a protein correlates with its size (R = 0.88). All the analyzed proteins present at least one aggregation segment in which half of the residues are closer than 3 Å to the interface, and 96% of them have at least one aggregation region in which >85% of the residues are adjacent to the interface (Table 2 and Figure 8). This supports the idea that the physico-chemical determinants of aggregation and native self-assembly might overlap significantly and is consistent with the observation that in homodimers, identical monomer subunits tend to associate by hydrophobic interactions [92]. After protein synthesis and folding, monomers probably associate rapidly into native homodimers due to the high local concentration of identical polypeptide chains, thus avoiding prolonged exposure of hydrophobic, aggregation-prone regions to solvent. Interestingly, in heterodimers, in which monomers spend a larger part of their lifetime in a non-associated state, the presence of gatekeeper amino acids (Lys, Arg, Glu, Asp, and Pro) at the complex interface is much greater than in homodimers [92], probably to prevent self-association between identical monomers.

**Table 2:** Overlapping of aggregation-prone regions and interfaces in non-amyloidogenic eukaryotic homodimers.

PDB	Protein	Source	Length	Aggregation segments	Aggregation segments close to the interfase (>50%)^1^	Aggregation segments close to the interfase (>85%)^2^
1F17	Dehydrogenase	Homo sapiens	293	9	2	2
1DQT	Antigen	Mus musculus	117	8	4	3
1LR5	Auxin binding protein	Zea mays	160	7	4	1
1KSO	Calcium-binding protein A3	Homo sapiens	93	3	2	2
1EAJ	Coxsackie virus	Homo sapiens	124	4	2	1
1PE0	DJ-1	Homo sapiens	187	7	1	1
1JR8	Erv2 protein mitochondrial	Saccharomyces cerevisiae	105	5	2	2
1F4Q	Grancalcin	Homo sapiens	161	6	3	1
1DQP	Guanidine phosphoribosyltransferase	Giardia lamblia	230	10	3	2
3SDH	Hemoglobin	Scapharca inaequivalvis	145	5	2	2
2HHM	Hydrolase	Homo sapiens	272	11	4	3
8PRK	Inorganic pyrophosphatase	Saccharomyces cerevisiae	282	8	3	2
1QMJ	Lectin	Gallus gallus	132	5	1	1
1M6P	Phosphate receptor	Bos taurus	146	5	2	1
1MNA	Polyketide synthase	Streptomyces venezuelae	276	10	2	1
1F89	Protein YLC351C	Saccharomy cerevisiae	271	11	3	2
1LHP	Pyridoxal kinase	Ovis aries	306	10	3	1
1QR2	Quinone reductase type 2	Homo sapiens	230	9	5	1
3LYN	Sperm lysine	Haliotis fulgens	122	6	3	1
1SCF	Stem cell factor	Homo sapiens	116	4	1	0
1HQO	URE2 protein	Saccharomyces cerevisiae	221	8	3	3
1HSS	Alpha-amylase inhibitor	Triticum aestivum	111	3	2	2
1KIY	Trichodiene synthase	Fusarium sporotrichioides	354	12	4	4
1MI3	Xylose reductase	Candida tenuis	319	6	1	1
1LBQ	Ferrochelatase	Saccharomyces cerevisiae	356	12	3	2

1 More than 50% of the residues in the aggregation-prone region are at less than 3 Å from a residue located at the interface of the complex.

2 More than 85% of the residues in the aggregation-prone region are at less than 3 Å from a residue located at the interface of the complex.

During the revision of the present work, Vendruscolo and co-workers published a related study in which they used their algorithm Zyggregator to perform an extensive analysis of interfaces in protein-protein complexes [93]. Interestingly enough, they independently concluded that interface regions are more prone to aggregate than other surface regions. Also, in excellent agreement with our analysis on monomeric soluble proteins, they found that charged residues frequently disrupt hydrophobic patterns at interfaces and that regions of negative aggregation propensity tend to surround aggregation-prone regions, which suggests that monomeric and native oligomeric proteins have evolved similar strategies to prevent misassembly. In our study, the analyzed eukaryotic proteins were randomly selected from a dataset of non-redundant homodimers [92], without any previous knowledge of their 3D-structures. Interestingly enough, the aggregation-prone sequences near to the dimer interface are located in α-helices in ∼70% of the cases (Figure 8). This is in clear contrast with their location in globular amyloidogenic polypeptides, where they reside mainly in preformed ß-strands. Although the sample is not statistically significant, this observation might suggest that natural selection is acting against the presence of amyloidogenic ß-strands at homodimers interfaces. It is attractive to propose that, as shown here for amyloidogenic proteins, mutations at these protein interfaces and specifically at protective locations might lead to loss of function or toxic phenotypes in a significant number of, yet undescribed, human polypeptides.

### Conclusions

In the present work, we have used computational tools to predict aggregation-prone regions and interaction sites in globular proteins related to depositional diseases and non-pathogenic polypeptides. From the comparison of the predictions with the structural and experimental data, it appears that protein-protein interaction surfaces and regions with high aggregation propensity overlap significantly in the quaternary structure of proteins.

The proximity and coincidence of protein-protein interfaces and aggregation-prone regions suggests that the formation of native complexes and the aggregation of their monomeric subunits probably compete in the cell. This implies that the molecular machinery that performs the vast array of cellular functions and the aggregates that might interfere with these functions promoting cell stress or even cell death are sustained by similar molecular contacts. It is likely that the specificity of native protein interfaces in protein complexes has evolved to minimize anomalous interactions and therefore detrimental protein aggregation reactions. In this sense, Vendruscolo and co-workers have recently identified disulfide bonds and salt bridges as specific interactions that can stabilize aggregation-prone interfaces in their native conformations in oligomeric proteins [93]. However, the balance between functional and aberrant self-assembly appears to be so delicate that point mutations that affect the interface or the stability of the complex, promoting a higher dissociation rate, usually lead to the formation of toxic aggregates, either through direct assembly of newly exposed aggregation-prone regions or by local unfolding of protein segments previously stabilized in the native structure of the complex.

Overall, the present analysis provides a rational to understand how globular proteins aggregate under physiological conditions, where they posses an initially folded and cooperatively sustained conformation and extensive denaturation is not expected to occur. The data strongly suggest that the stabilization of the interface in multimeric proteins, as in the case of TTR, SOD1, or LC immunoglobulins, and/or the blocking of conformational fluctuations and exposed amyloidogenic regions through the formation of new interfaces with other protein molecules, as in the case of lysozyme or Aß peptide, might be important strategies to delay the onset or slow the progress of conformational diseases caused by globular proteins.

The observed association between the failure to attain a native interface and the build up of harmful aggregates suggests that the range of genetic human diseases which ultimately might originate from the conversion of a soluble globular protein into toxic assemblies could be much larger than previously thought. Approaches combining the prediction of aggregation-prone regions from the linear protein sequence with the analysis of real or predicted protein interfaces in the 3D-structure might provide a means to identify physiologically and therapeutically relevant amyloidogenic sequences in the proteins linked to such disorders.

## Methods

### Prediction of Aggregation-Prone Regions

Aggregation-prone regions in the studied proteins were predicted using the primary sequence as input and a consensus of the output of four different available methods. The first algorithm we used is TANGO (http://tango.crg.es/). TANGO is based on the physico-chemical principles underlying ß-sheet formation, extended by the assumption that the core regions of an aggregate are fully buried [38]. The second algorithm employed was AGGRESCAN (http://bioinf.uab.es/aggrescan/). AGGRESCAN is based on the use of an aggregation-propensity scale for natural amino acids derived from *in vivo* experiments [40]. The third method, developed by Galzitskaya and co-workers, is based on the use of a packing density scale for natural amino acids and on the assumption that amyloidogenic regions are highly packed in the fibrillar structure [41]. The last approach was developed by Zhang and co-workers (ftp://mdl.ipc.pku.edu.cn/pub/software/pre-amyl/). It uses the microcrystal fibrillar structure of the prion hexapeptide NNQQNY [94] as a template and a residue-based statistical potential to identify amyloidogenic fragments of proteins [43]. All analysis was performed using the default parameters for each employed algorithm. In the present work, a sequence stretch in the analyzed proteins should comprise a minimum of five consecutive residues and be positively predicted by at least two of the above-mentioned methods to be considered an aggregation-prone region.

### Prediction of Protein-Protein Interaction Sites

Interaction residues were predicted using the monomeric three-dimensional crystal structure of each of the studied proteins as input and a consensus of the output of three different algorithms. The first approach used to predict interaction surfaces was the Optimal Docking Area (ODA) method (http://www.molsoft.com/oda), which identifies continuous surface patches with optimal docking desolvation energy based on atomic solvation parameters adjusted for protein-protein docking [32]. Only the top ten ODA hot spots were considered. The second method we used was SHARP^2^ (http://www.bioinformatics.sussex.ac.uk/SHARP2). SHARP^2^ calculates multiple parameters for overlapping patches of residues on the surface of a protein. It considers the solvation potential, hydrophobicity, accessible surface area, residue interface propensity, planarity, and protrusion. Parameter scores for each patch are combined, and the patch with the highest combined score is predicted as a potential interaction site [31]. The patch size was selected by considering the interacting partner to be an identical protein, and only residues in the best-scoring patch were considered. The last algorithm used was InterProSurf (http://curie.utmb.edu/). This method is based on solvent-accessible surface area of residues in isolated proteins, a propensity scale for interface residues, and a clustering algorithm to identify surface regions with residues of high interface propensities [33]. Only the first five clusters were considered. All analysis was done using the default parameters for each algorithm. In the present work, a residue in the surface should be identified as at least by two of the above mentioned approaches to be considered an interaction site.

### Evaluation of Interface Proximity

To evaluate whether the proximity of an aggregation-prone region to a given real interface is specific or the sequence stretch is as close to any other patch of the same size in the protein surface, we have defined the Interface Proximity Index: IPI

*IPI = 1-(SP/IP)*

*IP* = *Interface Proximity* = *nR/nHS*



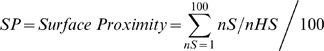



*nR* = number of residues in the aggregation-prone region at less than 3 Å from the interface.

*nHS* = number of residues in the aggregation-prone region.

*nS* = number of residues in the aggregation-prone region at less than 3 Å from a randomly chosen protein surface that does not include the interface.

Each random surface was generated by an aleatory selection of a number of solvent exposed residues equal to the number of residues constituting the real interface. One hundred random surfaces were generated for each aggregation-prone region analyzed.

Solvent-accessible and buried residues in the monomeric complex subunits where identified using the PISA server at the European Bioinformatics Institute (http://www.ebi.ac.uk/msd-srv/prot_int/pistart.html).

An *IPI*≤0 indicates that the aggregation-prone region is equally or less close to the interface than to the rest of the surface. An *IPI*>0 indicates that the aggregation-prone region is closer to the interface than to the rest of the surface, e. g., an *IPI* = 0.5 indicates that the aggregation-prone region is half as far from the interface than from the rest of the surface. The maximum value for *IPI* is 1.

Figures were were generated with the Swiss-PDB viewer program (http://spdbv.vital-it.ch) and rendered with POV (Persistance of Vision).
